# Sickle cell trait and priapism: a case report and review of the literature

**DOI:** 10.1186/1757-1626-1-429

**Published:** 2008-12-30

**Authors:** Brian F Birnbaum, Joseph J Pinzone

**Affiliations:** 1 The Ohio State University, The Ohio State University, 1654 Upham Drive, 207 Means Hall, Columbus, OH 43210, USA

## Abstract

**Background:**

A 32 year-old African-American man presented to our institution after attempting suicide via ingestion with quetiapine. He had reported a history of several days of substance abuse with alcohol, cocaine and marijuana related to a partying binge. Following this, his partner removed him from his residence resulting in a suicide attempt. During hospitalization the patient developed priapism, a condition he had not experienced before.

**Case presentation:**

Given this was his first time with priapism, an extensive work-up revealed the patient had previously undiagnosed sickle cell trait, which we postulate to have been a significant factor in his development of acute priapism. Sickle cell trait is considered to be a generally benign condition except for a few rare complications under more demanding physical conditions. However, upon reviewing the literature on the association of sickle cell trait with priapism, we believe this may not be the case. Case reports and small series that appeared in the 1960s and 1970s indicated an association between priapism and sickle trait. Little has been reported recently, and the general teaching regarding sickle cell trait does not include this information. However, one case was reported with the use of phosphodiesterase-5 (PDE-5) inhibitors and the development of priapism in a patient with sickle cell trait. These medications are now first line treatment in erectile dysfunction. They act by enhancing nitric oxide (NO) production leading to relaxation of smooth muscle in the corpora cavernosa and penile arteries.

**Conclusion:**

Priapism was not reported in the initial studies of these medications. Further review of the literature indicates this may be a complex relationship. Interestingly, PDE5 inhibitors also have been postulated to be protective in sickle cell disease and perhaps also sickle cell trait because priapism might be caused by reduced NO availability. In this article, we examine the evidence linking sickle cell trait to priapism, explore the implications of PDE5 use, particularly in the setting of sickle cell trait, and propose that teaching about sickle cell trait include a discussion of priapism risk.

## Case presentation

A 32 year-old African-American man with a history of substance abuse, type-2 diabetes mellitus and hypertension presented with mental status changes. He reported using cocaine, alcohol and marijuana for several days, and then attempted suicide by ingesting approximately 7 – 9 quetiapine tablets. He was treated with charcoal, rapid sequence intubation with succinylcholine, etomidate, and vecuronium, midazolam and propofol for sedation. Complete blood count, serum chemistries, blood cultures, ammonia level, liver function tests and head computed tomography were normal except for potassium 2.9 and creatinine of 1.2. Blood level for alcohol 0.088, acetaminophen < 0.1, and salicylate < 4. Electrocardiogram showed a heart rate of 125 bpm and QTc interval of 500 milliseconds. The patient developed a persistent erection during insertion of a Foley catheter, with no history of priapism. Medications prior to admission included quetiapine, amlodipine, atorvastatin, quinapril, glipizide, duloxetine and divalproex sodium, though he was not taking them regularly. Exam was notable for blood pressure 180/101, heart rate 103, respiratory rate 18, and moderate distress secondary to penile pain. Penis was turgid with pallor, and testes were descended bilaterally and normal in size and consistency. Phenylephrine was injected into the penis without relief. Subsequently, approximately 800 mL of blood was aspirated from the corpora cavernosum, but turgidity remained unchanged. The following day phenylephrine injection and aspiration of blood were repeated, with a substantial decrease in turgidity, though a partial erection remained for several more days with an estimated 30% erection at discharge. Work-up included a hemoglobin (Hb) electrophoresis which demonstrated HbS 34.8%, HbA 60.5%, and HbA_2 _4.2%, consistent with sickle cell trait.

## Erectile function and sickle cell trait

Sickle cell trait involves one normal beta-globin chain and one HbS chain, and is typically a benign condition. In general, priapism is not believed to be associated; however, we suspect that sickle cell trait is an important predisposing factor for priapism.

A normal erection occurs with increased blood flow to the corpera cavernosa and reduced outflow. Potential mechanisms for vascular occlusion in sickle cell disease include microvascular occlusion by sickled erythrocytes, vascular intimal hyperplasia, thrombosis and thromboembolism, fat embolism and vasospasm or failure of compensatory vasodilation. Microvascular occlusion is most important, and is caused by deoxygenated irreversibly sickled erythrocytes that have increased viscosity and endothelial adherence. Some erythrocytes are believed to polymerize even at normal arterial oxygen levels, and red blood cell dehydration may also play a role. Increased endothelial adherence may relate to sialic acid abnormalities, oxidative membrane damage, loss of membrane phospholipid asymmetry and binding to plasma proteins. Vascular intimal hyperplasia is a cause of cerebral vascular accidents in sickle cell disease, and it may be a factor in priapism. [1] "Low flow" priapism may result from decreased penile venous NO concentration resulting in vasoconstriction, reduced blood flow, deoxygenation and sickling. [2] Sickling promotes a vicious cycle resulting in reduced blood flow and worsening deoxygenation.

A series of patients with priapism published in 1961 included a single case of priapism in a 39 year-old with sickle cell trait. The patient was treated with anticoagulation, ice and sedation initially, followed by aspiration and irrigation on the third day. [3] Another case involved a 27 year-old man with 24 hours of priapism after vigorous coitus. Treatment with anticoagulation and anesthesia was unsuccessful, and was followed by evacuation of the corpora cavernosa. [4] Subsequent reports include a 20 year-old male who sustained an accidental kick to the shaft of his penis following intercourse with his wife, and after 48-hours sought medical attention. Cold showers and ethyl chloride spray did not work. Blood work indicated HbA 54.9%, HbA_2 _4.5%, HbS 39.6%, and HbF 1.0% consistent with sickle cell trait. Blood was evacuated from the corpora and showed 95% sickling. Priapism returned and whole blood transfusions showed no improvement. Bilateral greater saphenous vein to corpus cavernosum shunts resulted in slight improvement. A continuous pressure dressing was applied, resulting in a 20% decrease in size, but there was little improvement over the next 17 days. Continuous vacuum drainage led to complete resolution of the erection over a week. Within 25 days postoperatively two spontaneous nocturnal erections associated with orgasm were experienced, and 10 months following discharge the patient reported having completely satisfactory intercourse despite only partial erections. [5]

A 37 year-old man presented with acute priapism for 36 hours following sexual activity. [6] Masturbation did not alleviate his symptoms. He had two similar episodes previously. Hemoglobin electrophoresis indicated HbA 57.3% and HbS 42.7%. He was placed on bedrest and sedated, and penile ice packs were applied with no significant improvement. On hospital day eight bilateral aspiration of blood from the corpus cavernosa and irrigation with heparinized saline had an immediate partial response, but a return to full erection within two hours. Intravenous infusion of low molecular weight dextran for five days following the failed aspiration had no response. The patient was then offered a saphenous vein to corpus cavernosum shunt which resulted in flaccidity during the procedure, but continuation of the erection for six days postoperatively, and then a return to flaccidity.

A 14 year-old boy with priapism of seven days duration was found to have sickle cell trait and was treated initially with aspiration with a large bore needle, then with a sapheno-crural shunt. Three days following the shunt procedure, there was loss of the skin of the penile shaft and glans that was treated with split thickness skin grafting. The treatment resulted in 80% flaccidity; however, there was a left shift of the penile shaft secondary to fibrosis, thought to be caused by the shunt. [7]

Of a series of 23 cases of priapism, one had sickle cell trait; a 34 year-old with forty hours of turgidity following intercourse, who had an episode three months earlier treated with fibrinolysin (a bovine extract that causes fibrinolysis), aspiration and anticoagulation. [8] His current episode was treated with analgesics, heparinized saline followed by a sapheno-corporeal anastomosis. A blood pressure cuff and pressure dressing was applied. Catheterization took place on the second day along with ongoing anticoagulation, and the erection resolved within five days of this procedure. He then had satisfactory intercourse with one prolonged erection eleven months postoperatively that lasted six hours and spontaneously resolved.

Perhaps the most convincing evidence of a link between priapism and sickle cell trait was a report of 46 cases where 7 of the 24 (29%) African-Americans had sickle cell trait. Of the patients with sickle cell trait, four were treated with aspiration, two medically, and one with a shunt. [9]

## Erectile function and medications

Various medications have been used for erectile dysfunction, however the most commonly used are the phosphodiesterase-5 (PDE5) inhibitors sildenafil, tadalafil and vardenafil. [10] These medications are thought to enhance production of NO, which leads to the relaxation of the smooth muscle in the corpora cavernosa and penile arteries. There were no reports of priapism with these medications during initial studies; however, subsequently a report of acute priapism with sildenafil in a patient with sickle cell trait raised some concern. [11]

In contrast, one group postulates that PDE5 inhibitors may actually protect against priapism in sickle cell disease, because priapism might be a manifestation of reduced NO availability. [2] Therefore, it seems logical to also explore whether arginine or inhaled NO might also be beneficial. In fact, one group proposed that self-management of priapism might work best. The report cites a 38 year-old man with sickle cell trait and recurrent priapism who treated prolonged erections with self-injection of metaraminol for a period of 12 years, injecting as often as once every other day. [12] Over this period, he did not report to the emergency room, has not had any systemic side effects, and has not shown any signs of fibrosis at the injection site.

The patient had taken a number of medications and illicit drugs prior to receiving medical care, and had a number of medications administered while receiving care. Of the medications that he reported taking or were given to him, quetiapine, propofol, cocaine, alcohol, and marijuana have been associated with priapism.

## Discussion

There are multiple possible etiologies of the patient's priapism; however, it appears that sickle cell trait was likely a predisposing factor and we believe that this represents an important public health issue. In light of the explosive use of PDE5 inhibitors, we believe that studies need to investigate the potential association between sickle cell trait and priapism, and how treatments for erectile dysfunction play into this relationship. Moreover, the classical teaching that sickle cell trait is a benign condition should be reconsidered.

## Consent

Written informed consent was obtained from the patient for publication of this case report and accompanying images. A copy of the written consent is available for review by the Editor-in-Chief of this journal.

## Competing interests

The authors declare that they have no competing interests.

## Authors' contributions

BFB was the primary person responsible for the writing of the manuscript

JJP had full access to all of the data in the study and takes responsibility for the integrity of the data and the accuracy of the data analysis
